# Operationalizing two UAE nursing education policy frameworks: a cross-walk for curriculum, assessment, and continuous program improvement

**DOI:** 10.3389/fmed.2026.1940112

**Published:** 2026-10-21

**Authors:** Fatma M. Ibrahim, Ghada Shahrour, Ibrahim Al Faouri

**Affiliations:** 1 RAK College of Nursing, RAK Medical and Health Sciences University, Ras Al Khaimah, United Arab Emirates; 2 Faculty of Nursing, Mansoura University, Mansoura, Egypt; 3 Faculty of Nursing, Jordan University of Science and Technology, Irbid, Jordan

**Keywords:** curriculum mapping, nursing education, policy cross-walk, practice readiness, program evaluation, program of assessment, quality improvement, United Arab Emirates

## Abstract

**Background:**

The UAE Professional Practice Framework for Bachelor of Sciences in Nursing (BSN) Graduates and the national Outcome-Based Evaluation Framework (OBEF) address related but distinct questions. The first defines expected graduate competence; the second evaluates program and institutional outcomes. Programs need a practical method for using both documents together without treating institutional indicators as evidence of an individual learner's competence.

**Purpose:**

To develop a domain-level policy cross-walk and explain how nursing programs can use it to (1) create or refine curricula, (2) create or refine an integrated program of assessment, and (3) evaluate program quality and effectiveness.

**Approach:**

The three-author team conducted a structured analysis of the two national policy documents. The seven BSN domains and 38 standards were retained verbatim at domain and standard level. The team translated each domain into an observable graduate outcome, identified candidate direct evidence, and linked aggregated evidence to OBEF indicators only when the intended inference was compatible. Linkages were reviewed iteratively against predefined rules and resolved by consensus.

**Results:**

The cross-walk distinguishes three evidence roles: direct learner-performance evidence for feedback and progression; corroborating transition and stakeholder evidence; and distal program or institutional indicators. The combined model aligns the two policy documents and organizes application around curriculum, an integrated program of assessment, and program evaluation. Three tables provide the domain-level cross-walk, an evidence-to-decision guide, and a minimum institutional implementation record.

**Conclusion:**

The cross-walk offers an auditable starting architecture rather than a new national framework or validated assessment system. Local stakeholder review, staged piloting, monitoring of feasibility and equity, and documented continuous-improvement cycles are required before high-stakes use.

## 1 Introduction

The United Arab Emirates (UAE) now has two national policy documents that are particularly consequential for Bachelor of Science in Nursing (BSN) programs. The UAE Professional Practice Framework for Bachelor of Sciences in Nursing Graduates specifies seven interdependent domains and 38 competency standards, describes development across four levels of Miller's pyramid, and proposes assessment approaches (1). The Outcome-Based Evaluation Framework (OBEF), Version 11.5, evaluates higher education through 24 indicators distributed across employment outcomes, learning outcomes, industry collaboration, research outcomes, reputation, and community engagement (2). Twenty-two of these indicators apply at program level as well as institutional level.

The documents are complementary, but they operate at different levels. The BSN framework asks what a graduating nurse should be able to demonstrate. OBEF asks how well a program or institution performs on defined educational, employment, partnership, research, reputation, and community outcomes. The distinction is important because curriculum coverage, employment, satisfaction, licensure, or event counts cannot establish that an individual learner can provide safe nursing care. Conversely, learner assessment results alone cannot explain program effectiveness, implementation quality, or wider institutional contribution.

The practical challenge is therefore not simply to place both frameworks in the same table. Programs need an explicit chain linking each national nursing standard to an observable program outcome, planned learning opportunities, appropriate evidence, learner decisions, aggregated program findings, and quality-improvement action. Without this chain, a curriculum map may become a compliance checklist, assessment may remain fragmented across courses, and OBEF metrics may be interpreted beyond the claims they can support.

This Policy and Practice Review develops a domain-level cross-walk for operational use. Its aim is to help nursing programs use the two national frameworks together to: (1) create or refine curricula; (2) create or refine an integrated program of assessment; and (3) evaluate program quality and effectiveness. The proposed cross-walk is an interpretive policy tool. It is not an official CAA instrument, a new competency framework, or a validated assessment system, and it does not prescribe universal instruments, observation counts, cut scores, or progression thresholds.

## 2 Policy documents and cross-walk method

This review used structured policy-document analysis and focused synthesis rather than a systematic literature review. The two focal documents were purposively selected because they define the national expectations that UAE BSN programs must operationalize: the 2022 professional practice framework and the 2026 OBEF University Guidebook, Version 11.5 (1, 2). Peer-reviewed nursing and health-professions education literature was used selectively to inform practical safeguards for curriculum mapping, clinical assessment, longitudinal evidence, and program evaluation. No claim of exhaustive evidence coverage or intervention effectiveness is made.

The analysis was completed by the three-author team. FMI extracted the seven domains, 38 official standards, developmental expectations, and suggested assessment approaches from the BSN framework. FMI also extracted OBEF pillars, indicators, level of application, and stated purpose. GS and IF independently reviewed the proposed linkages for fidelity to the two documents, clarity of the educational claim, and risk of overinterpretation. The authors revised the cross-walk iteratively and resolved differences through discussion and consensus. No external panel, learner group, employer panel, or regulator participated; the resulting linkages therefore require stakeholder review and empirical testing.

Five decision rules governed the cross-walk. First, official BSN domain and competency-standard names were preserved. Second, a learner-readiness claim required direct evidence of observable performance; course coverage or distal outcomes were not accepted as substitutes. Third, OBEF indicators were linked according to their stated program or institutional purpose, not relabeled as measures of individual competence. Fourth, each linkage had to specify the decision it could reasonably inform. Fifth, the cross-walk had to be usable for curriculum review, assessment planning, and a documented improvement cycle.

The team applied these rules in four analytic steps. It first translated each BSN domain into a concise, observable graduate-level program outcome. It then identified integrated practice tasks and candidate direct evidence capable of sampling that outcome. Next, evidence was classified by role: direct learner-performance evidence, corroborating transition or stakeholder evidence, or distal program and contextual indicators. Finally, aggregated findings were connected to relevant OBEF indicators and to a defined program-evaluation question. The method produced the combined architecture shown in Figure 1 and the operational cross-walk in Tables 1–3. No learners, institutional records, or patient data were used; ethics review was not applicable. The authors acknowledge the use of AI tools to assist with English-language editing and manuscript organization during revision. The tool was not used to select or extract policy content, determine cross-walk linkages, generate or modify data, or conduct analyses. The authors checked all AI-assisted text and visual content for accuracy, fidelity to the source documents, citation validity, and originality, revised the outputs critically, and accept full responsibility for the manuscript.

**Figure 1:**
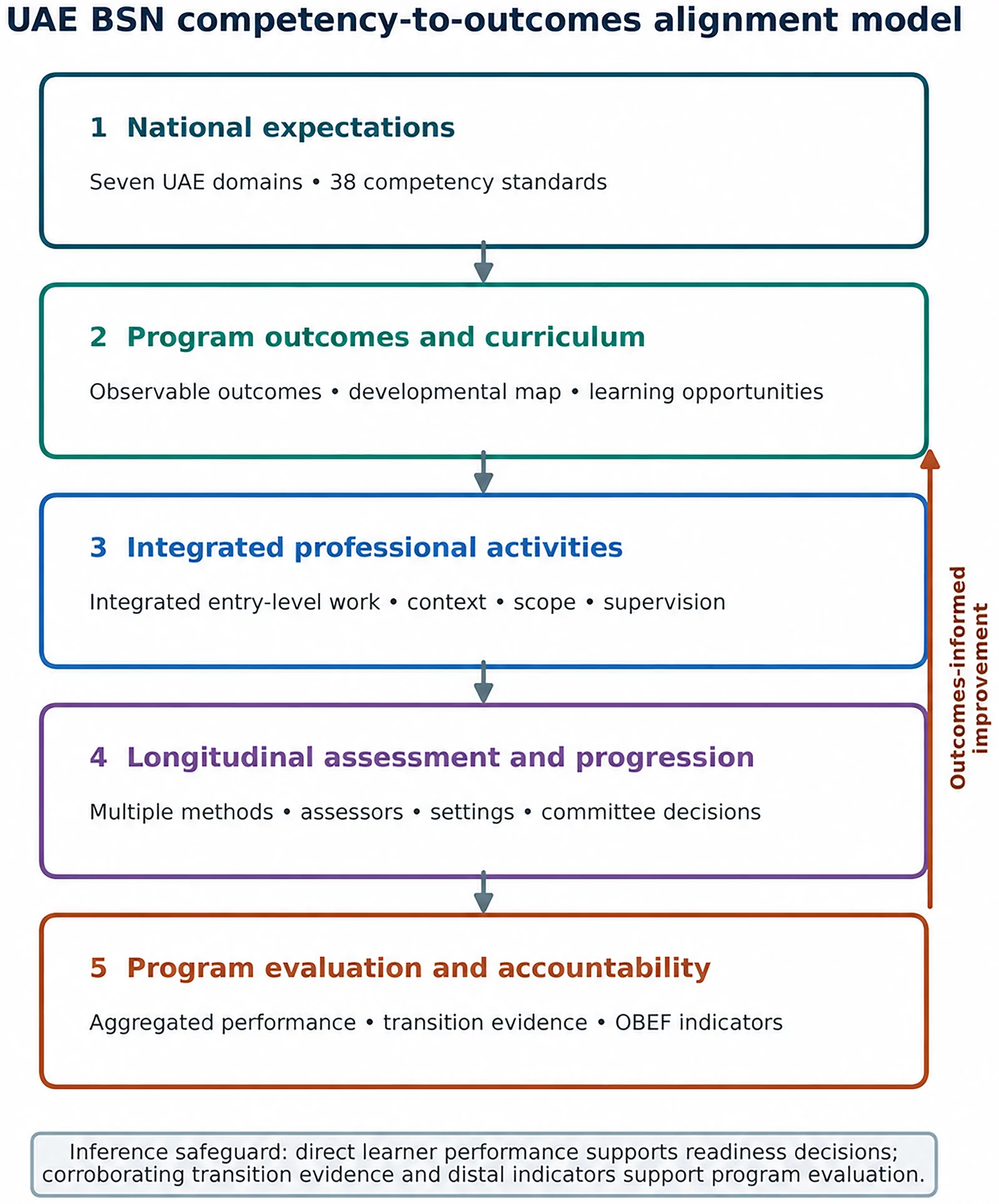
Combined use of the UAE BSN professional practice framework and the outcome-based evaluation framework. The two policy documents provide complementary lenses. The combined cross-walk preserves the distinction between direct learner assessment and aggregated program evaluation and supports three institutional applications: curriculum development or refinement, an integrated program of assessment, and program evaluation with continuous improvement.

**Table 1:** Domain-level cross-walk from national BSN expectations to learner evidence and program-evaluation use.

UAE BSN domain and official standards	Observable graduate outcome	Integrated practice tasks	Candidate direct learner evidence	Aggregated program-evaluation use and relevant OBEF signals
Person-Centered Care: Holistic Care; Clinical Reasoning and Judgement; Therapeutic Interventions; Safe & Quality Care; Care Transition and Continuity	Integrate assessment, clinical judgment, evidence, and patient preferences to plan, deliver, evaluate, and coordinate safe care across the lifespan.	Holistic assessment; care planning; therapy administration and monitoring; recognition of deterioration; care transition.	Repeated clinical observations; multi-patient simulation; OSCE; review of care plans and documentation.	Identify gaps in reasoning, safety, continuity, and opportunity; triangulate with OBEF 2.1, 2.3–2.5 and, cautiously, 1.2.
Ethical and Legal Practice: Ethical and Moral Practice; Legal Practice; Accountability; Professional Development; Cultural Competency; Advocacy	Practice within ethical, legal, and professional boundaries while demonstrating accountability, cultural responsiveness, advocacy, and development.	Consent and confidentiality; safeguarding; advocacy; scope recognition; escalation of ethical or legal concern.	Direct observation; ethics cases; professionalism narratives; documentation and portfolio evidence.	Examine patterns in professionalism, incidents, remediation, and equity; triangulate with OBEF 2.1, 2.3–2.4 and 5.2.
Communication and Collaboration: Effective Communication; Effective Therapeutic Relationship; Interdisciplinary Communication; Teamwork; Positive Work Environment	Communicate responsively with people, families, and teams and contribute to respectful, coordinated interprofessional care.	Therapeutic communication; education; shared decisions; handover; interprofessional planning and evaluation.	Communication OSCE; handover observation; team simulation; repeated clinical assessment.	Identify communication and teamwork gaps across courses and sites; triangulate with OBEF 2.3–2.4 and 3.3.
Research & Evidence-Based Practice: Scholarly Practice; Evidence-Based Practice; Knowledge Integration; Innovation and Entrepreneurship Practice	Formulate practice questions, appraise and integrate evidence, and contribute to quality improvement and context-appropriate innovation.	Evidence appraisal; response to a clinical question; quality-improvement work; responsible innovation.	Critical appraisal; evidence-based practice project; quality-improvement proposal; oral defense.	Evaluate evidence use and project quality; triangulate with OBEF 2.1, 4.1, 4.3 and 4.4 without equating output counts with competence.
Community and Public Health: Health Education; Health Promotion; Illness Prevention; Community-Based Practice; Disaster Management; Public Health and Wellbeing	Assess population needs and implement culturally responsive health-promotion, prevention, community-care, and emergency-preparedness interventions.	Community assessment; health education; prevention; disaster preparedness; population intervention.	Community assessment; observed education; fieldwork; disaster simulation; project evaluation.	Examine reach, partner feedback, learner performance, and population signals; connect cautiously with OBEF 6.1–6.2.
Leadership and Management: Quality Management; Problem Solving Decision Making; Risk and Crises Management; Autonomy; Empowerment & Delegation; Conflict Management; Resource Management	Prioritize, coordinate, and evaluate care; delegate within scope; manage risk and resources; and contribute to quality and safety.	Multi-person prioritization; delegation; risk and crisis management; resource stewardship; conflict response.	Multi-patient simulation; clinical observation; delegation/prioritization exercise; quality project.	Detect gaps in prioritization, risk management, and clinical opportunity; triangulate with OBEF 2.3–2.4 and 3.1–3.3.
Informatics & Technology: Health Information Integration; Informatics-Guided Care; Electronic Documentation; Artificial Intelligence Utilization; Tele-Health Care	Use information systems, digital technologies, artificial intelligence, and telehealth safely, ethically, and critically to support care.	Electronic documentation; data-informed care; telehealth; critical appraisal of artificial intelligence; digital professionalism.	Electronic health-record task; documentation audit; digital-health OSCE; artificial-intelligence appraisal and safety scenario.	Evaluate documentation quality, technology-related safety, digital professionalism, and site capability; connect with OBEF 2.1 and 2.3–2.4.

Official domain and competency-standard names are reproduced from (1). Outcomes, tasks, evidence sources, and program uses are interpretive proposals for local review and validation. OBEF linkages are program-level signals, not measures of individual competence. OSCE, objective structured clinical examination.

**Table 2:** Evidence roles, decision uses, and OBEF connections.

Evidence role	Examples	Decision supported	OBEF connection	Misuse to avoid
Direct learner-performance evidence	Repeated clinical observation; OSCE; simulation; reasoning tasks; products of care; projects.	Individual feedback, progression, remediation, and graduation; aggregated curriculum and assessment diagnosis.	Informs the program's internal evidence base; OBEF 2.1 reviews assessment quality rather than certifying individual competence.	Treating course coverage, hours, one encounter, or one total score as proof of overall readiness.
Corroborating transition and stakeholder evidence	Clinical-preceptor and employer feedback; licensure; graduate transition; early-career evidence.	Whether program-level patterns appear to extend into placement, licensure, or employment.	OBEF 1.1–1.2, 2.3–2.5, and 3.1.	Using satisfaction, employer ratings, licensure, or employment as a substitute for direct learner assessment.
Distal program and contextual indicators	Retention; student experience; placement participation; partnerships; research; accreditation; community engagement.	Program reach, context, partnership, productivity, reputation, and societal contribution.	OBEF 2.2, 2.6, 3.2–3.4, 4.1–4.4, 5.1–5.4, and 6.1–6.2 at both program and institutional levels; 4.5–4.6 at institutional level only. Check applicability in the current guide.	Claiming that favorable program or institutional metrics establish safe clinical performance or curricular causation.

Indicator names and applicability should be checked against the current official OBEF guide at the time of reporting (2).

**Table 3:** Minimum institutional record for operationalizing the cross-walk.

Required field	Operational question	Minimum record
National requirement	Which official domain and standard are addressed?	Verbatim domain/standard; policy version.
Program outcome	What integrated performance should the graduate demonstrate?	Observable outcome; scope and context.
Development	How does performance progress across the program?	Expected level by semester/year; prerequisite learning.
Learning opportunity	Where can learners acquire and practise the performance?	Course, placement/simulation/community context, responsible owner.
Direct evidence	What evidence will demonstrate the outcome?	Method, task, rater, timing, authenticity, and safety-critical behaviors.
Sampling	Is evidence sufficiently representative?	Required contexts, occasions, rater groups, and approach to missing opportunity.
Learner decision	How will evidence be used?	Feedback, progression, remediation, graduation; decision authority and appeal.
Program signal	What will aggregated evidence tell the program?	Indicator, denominator, disaggregation, threshold, review interval.
OBEF connection	Which OBEF indicator is relevant and at what level?	Indicator number/name; program or institutional use; stated evidentiary boundary.
Improvement and closure	What action follows and how will improvement be verified?	Finding, action, owner, resources, timeline, target, closure evidence.

## 3 Cross-walk results: combining the two policy frameworks

Figure 1 shows the organizing logic of the manuscript. The BSN framework supplies the professional expectations that must remain visible in curriculum and learner assessment. OBEF supplies program and institutional indicators that broaden evaluation beyond learner performance. The cross-walk combines these lenses while maintaining an evidentiary boundary: direct assessment supports claims about individual learners, whereas aggregated assessment, transition, stakeholder, and contextual indicators support claims about program quality and improvement.

Table 1 presents the core domain-level cross-walk. The second column converts each national domain into an observable graduate outcome without changing the official domain or standard names. The third and fourth columns identify integrated practice tasks and candidate direct evidence. The final column identifies program-evaluation signals and relevant OBEF connections. These connections are intentionally not one-to-one equivalences. For example, OBEF employer feedback may corroborate a program-level communication concern, but it cannot replace repeated observation of a learner's communication in clinical or simulated care.

Table 2 clarifies the role of each evidence category. Direct learner-performance evidence is closest to the competence claim and is the principal basis for feedback, progression, remediation, and graduation. When aggregated and disaggregated, the same evidence can help identify curriculum, assessment, or clinical-placement weaknesses. Corroborating evidence tests whether patterns appear to persist during transition to practice. Distal indicators describe program reach, partnerships, productivity, reputation, and societal contribution. The value of the cross-walk lies in using these sources together while preventing metric substitution.

The cross-walk is deliberately domain-level. It provides a common architecture across institutions but does not claim that seven broad outcomes are sufficient for implementation. Each program must expand Table 1 to the level of all 38 standards and record the expected developmental level, responsible courses, learning opportunities, direct evidence, sampling plan, and decision use. Table 3 specifies the minimum fields required for that local implementation record.

## 4 Using the cross-walk to create or refine curriculum

Curriculum use begins with a complete, evidence-oriented map rather than a binary indication that a standard is present. Curriculum mapping can expose omissions, unnecessary repetition, weak sequencing, and standards that are taught but not assessed authentically (3, 4). The cross-walk makes the map decision-focused by requiring programs to state what graduates must demonstrate and what evidence will be available when progression or program-review decisions are made.

### 4.1 Build the local 38-standard map

For every standard, the curriculum committee should complete the fields in Table 3. The map should identify the course and semester in which the standard is introduced, developed, demonstrated, and integrated; the practice context; the expected level; the responsible academic and clinical partners; and the assessment evidence produced. A blank field indicates a genuine design gap. Repeated entries should be examined for purposeful progression rather than assumed to be beneficial because the content appears frequently.

### 4.2 Test sequence, authenticity, and opportunity

The committee should then ask whether learning opportunities increase in complexity and authenticity. Knowledge and guided practice should precede standardized demonstration, followed by repeated performance in patient-care or community settings when safe and feasible. Simulation is appropriate for rare, high-risk, or standardized situations, but it should be described accurately and designed according to recognized standards (5). Programs should also examine whether learners at different clinical sites have comparable opportunities to demonstrate the required outcomes.

Integrated tasks are useful curriculum anchors because nursing work rarely belongs to one domain. Recognizing deterioration, for example, combines person-centered assessment, clinical reasoning, communication, escalation, ethical accountability, teamwork, documentation, and technology use. The map should show where learners rehearse this integration rather than assuming that separate course-level assessments will automatically combine into practice-ready performance.

### 4.3 Convert findings into approved curriculum decisions

A map becomes useful only when findings lead to decisions. For each gap or redundancy, the committee should record the evidence, proposed action, owner, approval route, resource implications, implementation date, and closure criterion. Adding content is not the default solution. Programs may need to remove duplication, change the timing of an experience, increase supervised practice, revise an assessment, improve clinical-site coordination, or strengthen feedback. Curriculum change should remain proportionate to the problem identified and should preserve transparent documentation for later evaluation.

## 5 Using the cross-walk to create or refine an integrated program of assessment

An integrated program of assessment is a planned system in which multiple assessment methods are selected for complementary purposes and interpreted across courses, raters, contexts, and time. It is not a collection of unrelated tests and rating forms, nor is it the mechanical addition of scores. Programmatic approaches emphasize the educational value of frequent feedback and the deliberate aggregation of sufficiently rich information for consequential decisions (6–8).

### 5.1 Create a program-level assessment blueprint

The blueprint should begin with the outcomes and standards in the local cross-walk. For each outcome, it should specify the claim, developmental expectation, assessment purpose, methods, contexts, sampling requirements, rater groups, timing, feedback process, remediation pathway, and decision authority. Method choice should follow the intended performance. Written examinations can assess knowledge and selected reasoning processes; objective structured clinical examinations can standardize selected performances; simulation can sample uncommon or high-risk situations; and direct clinical observation captures authentic performance. Portfolios, products of care, projects, and multisource feedback add different evidence. No single method is sufficient for complex practice readiness (9, 10).

The blueprint should also reveal domain imbalance. Community and public health, evidence-based practice, leadership, and informatics often require observable products or performances and should not be represented mainly by written examinations. Communication and teamwork require repeated observation across different people and settings. Safety-critical activities require explicit behaviors and escalation expectations.

### 5.2 Aggregate evidence for feedback and progression

Each assessment event should contribute information proportionate to its quality and purpose. Lower-stakes observations should primarily generate timely feedback and identify evidence gaps. Higher-stakes decisions should draw on a planned body of evidence rather than one encounter, one rater, a total number of clinical hours, or a compensatory overall score. Reviews of nursing clinical assessment continue to identify variation in instruments and assessor preparation, underscoring the importance of shared standards and rater development (9). UAE evidence concerning objective structured clinical examinations similarly highlights the need for preparation, consistent processes, and attention to learner stress (11).

A progression committee should use a predefined protocol to review evidence completeness, longitudinal trajectory, performance across contexts, unresolved safety concerns, response to feedback, and relevant narrative comments. Non-compensatory safety rules should identify critical behaviors that cannot be offset by high scores elsewhere. Academic regulations should specify committee membership, conflicts of interest, remediation, reassessment, appeal, and documentation. Raters require shared interpretation of standards, practice with benchmark examples, and support for concise narrative feedback (12).

### 5.3 Monitor the assessment system

The program should monitor missing observations, unequal opportunities, rater patterns, site effects, narrative quality, decision consistency, remediation outcomes, learner experience, and workload. More data are not inherently better; excessive assessment burden can reduce participation and feedback quality (13). A parsimonious system samples the breadth and complexity of practice while collecting only evidence that has a defined educational or decision purpose. OBEF indicator 2.1 can inform external review of assessment quality, but the program still requires its own evidence that the assessment system produces useful, fair, and sufficiently representative information.

## 6 Using the cross-walk to evaluate program quality and effectiveness

Program evaluation asks whether the curriculum, assessment system, learning environments, partnerships, and support structures enable cohorts to achieve intended outcomes. The cross-walk provides a program theory: national standards are translated into outcomes; learning opportunities and assessments generate direct evidence; progression processes act on that evidence; and aggregated findings are examined with OBEF and other contextual indicators. This chain allows evaluators to identify where a weak result may have arisen rather than treating every unfavorable indicator as a curriculum failure.

### 6.1 Build a tiered dashboard

The dashboard should retain the three evidence categories in Table 2. The first tier contains aggregated direct performance by domain, standard, cohort, course, clinical site, and stage of the program. The second tier contains corroborating evidence, including clinical-preceptor judgments, employer feedback in placements and employment, licensure outcomes, and graduate transition data. The third tier contains distal program and contextual indicators, including retention, student satisfaction, placement participation, employment, research, accreditation, and community engagement. These data should be presented side by side rather than collapsed into one composite score.

Disaggregation is essential. Overall means can conceal site-specific opportunity problems, inconsistent rating, or differential progression among learner groups. Programs should predefine the categories used for equity monitoring, protect confidentiality, and avoid unstable comparisons based on very small groups. Patterns should be interpreted with denominators, missingness, time frames, and contextual information visible.

### 6.2 Investigate convergence and plausible causes

A program-level concern is more persuasive when independent evidence converges. Repeatedly weak handover observations, lower communication performance in standardized encounters, and consistent clinical-partner feedback would justify investigation of curriculum sequence, practice opportunity, rater expectations, and placement conditions. By contrast, lower employment may reflect labor-market conditions, licensure delays, or graduate choices and should not automatically be attributed to curriculum quality. OBEF indicators are valuable signals, but causal claims require targeted investigation.

Evaluation should distinguish implementation failure from design failure. If a planned clinical observation process is rarely completed, the immediate problem may be workload, usability, training, or site access. If the process is implemented as intended but does not improve feedback or decision quality, the design itself may require revision. Competency-based education is a complex intervention; interpreting outcomes without implementation evidence risks drawing the wrong conclusion (14).

### 6.3 Close and document the improvement cycle

Each priority finding should lead to a corrective or enhancement plan that states the baseline, analysis, action, owner, resources, timeline, monitoring measure, target, and closure rule. This structure is consistent with OBEF's emphasis on evidence-based corrective action and measurable improvement (2). A revised syllabus, new form, or faculty workshop documents activity, not effectiveness. Closure requires evidence that the intended opportunity, assessment quality, learner performance, transition outcome, or program indicator changed as expected.

The program should review the cross-walk at a defined interval and whenever national standards, regulations, clinical practice, or OBEF indicators change. Version control is important: committees should retain the policy version, approved mapping decisions, rationale for changes, and the evidence used to close actions. This converts the cross-walk from a one-time accreditation product into a living quality-assurance tool.

## 7 Governance and staged implementation

Implementation should be staged because the cross-walk affects curriculum, assessment, academic regulation, clinical partnerships, workload, information systems, and quality governance. Curriculum-renewal experience in nursing suggests that coordinated change is usually iterative and extends across several semesters (15). A steering group should include program leadership, educators representing all seven domains, assessment and quality specialists, learners, clinical partners, and data-governance expertise.

### 7.1 Four implementation phases

**Phase 1—Policy and curriculum audit**. Verify the current versions of both policy documents, populate the 38-standard map, and prioritize gaps that affect safety, progression, or required reporting.

**Phase 2—Pilot priority changes**. Begin with a small number of integrated, high-risk activities and representative clinical contexts. Train raters and test workflow, opportunity, feedback, and burden.

**Phase 3—Establish governance**. Approve progression protocols, evidence dashboards, remediation and appeal routes, data governance, and committee responsibilities.

**Phase 4—Evaluate, refine, and scale**. Review implementation and outcomes, correct weaknesses, document closure, and expand only when evidence supports feasibility and educational value.

### 7.2 Core safeguards

Before high-stakes use, programs should approve definitions, decision rights, evidence requirements, safety rules, remediation and appeal processes, data access, retention, and audit procedures. Digital portfolios may support longitudinal review, but access control and correction processes are essential because narrative comments can contain sensitive educational or clinical information. If algorithmic or artificial-intelligence functions summarize narratives or flag performance, their purpose, accuracy, bias, and human oversight require separate evaluation.

Piloting should examine feasibility, clinical opportunity, data completeness, response processes, rater consistency, narrative usefulness, learner and rater burden, differential effects, and unintended consequences. The appropriate scale is the smallest implementation capable of answering the priority question. Expansion should occur only after the program can show that the process is usable, governed, and educationally valuable.

## 8 Discussion

The principal contribution of this review is a simplified operational cross-walk that places two UAE policy documents in combination and organizes their use around three institutional functions: curriculum, an integrated program of assessment, and program evaluation. This structure responds to a practical policy problem. It shows how the BSN framework's professional expectations can remain visible from course design through learner decisions, while OBEF indicators are used at the program or institutional level for accountability and improvement.

The cross-walk also establishes a necessary evidentiary boundary. An individual learner's readiness must be supported by direct and sufficiently representative performance evidence. Employer feedback, licensure, employment, satisfaction, research, reputation, and community-engagement indicators can strengthen program evaluation, but they cannot replace that evidence. At the same time, aggregated learner performance is not interpreted in isolation: it is examined with implementation, transition, stakeholder, and contextual data to identify plausible causes and appropriate action.

This approach is compatible with wider nursing-education movement toward explicit competencies and observable progression, while avoiding the assumption that importing another curriculum-design or assessment framework is necessary for local implementation (16, 17). Its utility lies in the disciplined questions it requires: What national expectation is being addressed? What should the learner demonstrate? What evidence is collected, where, by whom, and for what decision? What does the aggregated pattern indicate about the program? What action was taken, and what evidence will demonstrate improvement?

The review has limitations. It is a purposive policy analysis and interpretive synthesis, not a systematic review, formal consensus study, or validation study. The three-author process improved transparency but does not establish content validity. The proposed outcomes, integrated tasks, evidence sources, and OBEF connections have not been tested with UAE learners, educators, clinical partners, employers, patients, communities, regulators, or assessment experts. Feasibility, cost, inter-institutional comparability, and effects on learner or patient outcomes are unknown. OBEF requirements may evolve, and several indicators are influenced by labor-market and institutional factors beyond curriculum quality. These limitations preclude immediate national standardization and define the agenda for stakeholder refinement, pilot implementation, and evaluation.

## 9 Conclusion

The UAE BSN Professional Practice Framework and OBEF can be used together without confusing their purposes. The proposed cross-walk links national nursing standards to observable outcomes and direct learner evidence, then connects aggregated findings to program evaluation and relevant OBEF indicators. Its three uses are concrete: refine curriculum, build an integrated program of assessment, and evaluate program quality through a documented improvement cycle.

For nursing programs, the immediate task is to complete the 38-standard institutional map, identify evidence gaps and inequities in opportunity, blueprint assessment across the program, and establish governance for progression and improvement decisions. The cross-walk should be treated as an auditable starting architecture. Stakeholder co-design, staged piloting, and evidence of feasibility, fairness, educational value, and consequences are required before high-stakes scale-up.
